# *Candida* Administration Worsens Cecal Ligation and Puncture-Induced Sepsis in Obese Mice Through Gut Dysbiosis Enhanced Systemic Inflammation, Impact of Pathogen-Associated Molecules From Gut Translocation and Saturated Fatty Acid

**DOI:** 10.3389/fimmu.2020.561652

**Published:** 2020-09-25

**Authors:** Wimonrat Panpetch, Vorthon Sawaswong, Prangwalai Chanchaem, Thunnicha Ondee, Cong Phi Dang, Sunchai Payungporn, Somying Tumwasorn, Asada Leelahavanichkul

**Affiliations:** ^1^Department of Microbiology, Faculty of Medicine, Chulalongkorn University, Bangkok, Thailand; ^2^Program in Bioinformatics and Computational Biology, Graduate School, Chulalongkorn University, Bangkok, Thailand; ^3^Department of Biochemistry, Faculty of Medicine, Chulalongkorn University, Bangkok, Thailand; ^4^Center of Excellence in Systems Biology, Chulalongkorn University, Bangkok, Thailand; ^5^Medical Microbiology, Interdisciplinary Program, Graduate School, Chulalongkorn University, Bangkok, Thailand; ^6^Translational Research in Inflammation and Immunology Research Unit, Department of Microbiology, Chulalongkorn University, Bangkok, Thailand

**Keywords:** intestinal *Candida*, obesity, high-fat diet, probiotics, cecal ligation and puncture, dysbiosis, gut leakage

## Abstract

Obesity induces gut leakage and elevates serum lipopolysaccharide (LPS), a major cell wall component of Gram-negative bacteria, through gut translocation. Because *Candida albicans* is prominent in human gut but not in mouse, *C. albicans*, a source of (1→3)-β-D-glucan (BG) in gut contents, was administered in high-fat diet (HFD)–induced obese mice at 1 week before sepsis induction by cecal ligation and puncture (CLP). As such, sepsis in *Candida*-administered obese mice was more severe than obese mice without *Candida* as determined by mortality, organ injury (liver and kidney), serum cytokines, gut leakage, endotoxemia, serum BG, and fecal Gram-negative bacteria (microbiome analysis). Mice subjected to CLP and fed a HFD, but not treated with *Candida* demonstrated a similar mortality to non-obese mice with more severe gut leakage and higher serum cytokines. *In vitro* experiments demonstrated that LPS plus BG (LPS + BG) induced higher supernatant cytokines from hepatocytes (HepG2) and macrophages (RAW264.7), compared with the activation by each molecule alone, and were amplified by palmitic acid, a representative saturated fatty acid. The energy production capacity of HepG2 cells was also decreased by LPS + BG compared with LPS alone as evaluated by extracellular flux analysis. However, *Lactobacillus rhamnosus* L34 (L34) improved sepsis, regardless of *Candida* administration, through the attenuation of gut leakage and gut dysbiosis. In conclusion, an impact of gut *Candida* was demonstrated by *Candida* pretreatment in obese mice that worsened sepsis through (1) gut dysbiosis–induced gut leakage and (2) amplified systemic inflammation due to LPS, BG, and saturated fatty acid.

## Introduction

Both sepsis, a syndrome of imbalance immune responses to pathogens, and obesity are major healthcare problems worldwide (1–4). Whereas obesity induces several chronic conditions such as diabetes, dyslipidemia, and cardiovascular disease (5), sepsis is a major cause of death in critically ill patients, mostly with chronic underlying diseases (3). Indeed, obesity is categorized as a sepsis comorbidity and an independent risk factor for death of patients in the intensive care unit (6, 7), at least in part, due to the enhanced inflammation caused by adipocytes and immune cells (8, 9). In addition, obesity and high-fat diet (HFD) cause gut dysbiosis, an alteration of bacteria and fungi in gut (10), that induces gut-permeability defect (gut leakage) leading to spontaneous endotoxemia (11). The impact of obesity on sepsis remains a controversy as obesity worsens sepsis severity through the induction of several metabolic abnormalities but beneficially restores energy preserve in the moribund stage of sepsis (12, 13). However, endotoxemia increased systemic inflammation and enhanced sepsis severity (14). Although endotoxemia from obesity implies the importance of intestinal Gram-negative bacteria as a source of intestinal endotoxin (lipopolysaccharide; LPS), the impact of *Candida albicans* which is the second most predominant gut organism (15) on obesity is still not clear. On the contrary, the impact of intestinal *C. albicans* in other models has been mentioned. For example, increased abundance of *C. albicans* in alcohol ingestion model and in patients enhances liver cirrhosis through direct activation of intestinal (1→3)-β-D-glucan (BG) which is a major component of fungal cell wall against hepatocytes (16–18). In addition, intestinal *C. albicans* is a source of BG in gut contents. In addition, BG from gut translocation amplifies the inflammatory property of LPS through the synergy of Dectin-1 and Toll-like receptor (TLR)-4 which are receptors of BG and LPS, respectively, in sepsis and several inflammatory models (19–24).

Interestingly, HFD also increases the abundance of *Candida* spp. in mouse feces, but the abundance of fecal fungi in mouse feces is not high enough to be detectable by culture (10). Indeed, *C. albicans* in mouse intestine are lesser than human intestine (25) as fungi in human stool are easily detectable by culture in comparison with mouse feces (26). Hence, oral administration of *C. albicans* is necessary to increase mouse fecal fungi. Mouse models with fecal *C. albicans* more closely resemble human conditions, at least in part, because of the interaction between gut organisms (gut dysbiosis) (27). As such, *C. albicans* induce gut dysbiosis in sepsis (28) and sepsis with obesity (29, 30) that might be associated with gut leakage (31). In addition, gut leakage in obesity (11), and sepsis (14) is attenuated by probiotics (32–36), including *Lactobacillus* spp. that could interfere with *Candida* growth (37). Moreover, *Lactobacillus* spp. attenuated gut dysbiosis in several animal models (27, 38). Hence, an obesity mouse model was performed with *C. albicans* pretreatment before cecal ligation and puncture (CLP) sepsis with an evaluation on a probiotic. Understanding the influence of gut fungi in sepsis with obesity might be beneficial in sepsis treatment.

## Materials and Methods

### Animals and Animal Model

**FIGURE 1:**
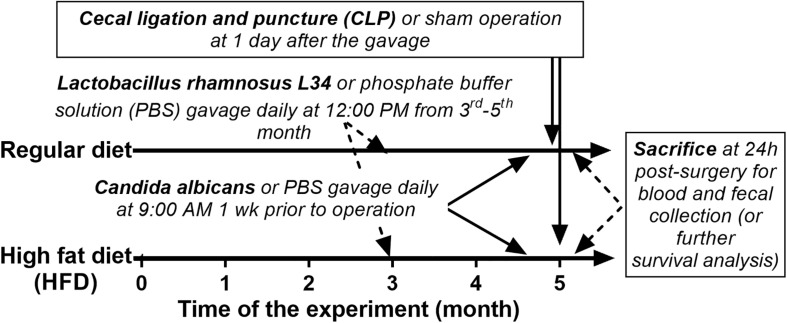
Schema of the experimental design is demonstrated.

The animal care and use protocol prepared according to the US National Institutes of Health standards was approved by the Institutional Animal Care and Use Committee of the Faculty of Medicine, Chulalongkorn University, Bangkok, Thailand (SST 04/2561). Male, 8-week-old C57BL/6 mice were purchased from the National Laboratory Animal Center, Nakhorn Pathom, Thailand. Mice in the regular diet group received standard laboratory chow containing fat (4.5% *w*/*w*), with energy content calculated at 3.04 kcal/g (Mouse Feed Food No. 082; C.P. Company, Bangkok, Thailand). Mice in the obese group were fed for 5 months with HFD containing fat, mostly from lard (60% *w*/*w*), with energy content calculated at 8.64 kcal/g following a publication (39). Schema of the experiments is demonstrated in Figure 1. At 3 months of the experiment, *Lactobacillus rhamnosus* L34 (L34) (38) at 1 × 10^9^ colony-forming units (CFU) in 0.5 ml phosphate buffer solution (PBS) or PBS alone were administered daily for 2 months before *Candida* administration. At 1 week before sepsis induction, *C. albicans* from American Type Culture Collection (ATCC90028; Fisher Scientific, Waltham, MA, United States) at 1 × 10^6^ CFU in 0.5 ml PBS or PBS alone were orally administered every 2 days to induce *Candida* in gut. At 24 h from the last dose of *Candida* or PBS, CLP or sham was performed following a publication with 10-mm cecal ligation and a 21-gage needle under isoflurane anesthesia (21). Fentanyl, 0.03 mg/kg in 0.5 ml of normal saline solution (NSS), was subcutaneously administered at post-operation and at 6 h later. Mice were sacrificed at 24 h post-surgery under isoflurane anesthesia with blood and organ collection. Ascending colon 1 cm from colon–cecal junction was snap frozen in liquid nitrogen and kept at -80°C before use. Feces from all parts of colon were combined and collected for microbiome analysis and fecal fungal burdens.

### Mouse Blood Sample Analysis and Gut Leakage Measurement

Several obesity parameters were determined after fasting for 12 h with free access to drinking water. Fasting glucose and triglyceride were measured by glucose colorimetric assay (Cayman Chemical, Ann Arbor, MI, United States) and triglyceride quantification kit (Sigma-Aldrich, St. Louis, MO, United States), respectively. The lipid profile was evaluated using assays of total cholesterol quantitation (Sigma-Aldrich), low-density lipoprotein cholesterol (LDL; Crystal Chem, Downners Grove, IL, United States), and high-density lipoprotein cholesterol (HDL; Crystal Chem). Renal injury and liver damage were determined by QuantiChrom Creatinine Assay (DICT-500; Bioassay, Hayward, CA, United States) and EnzyChrom Alanine Transaminase assay (EALT-100; BioAssay), respectively. Serum cytokine levels were determined by ELISA for mouse cytokines [tumor necrosis factor (TNF)-α, interleukin (IL)-6, and IL-10] (Invitrogen, Carlsbad, CA, United States). Gut leakage was determined by detection of fluorescein isothiocyanate (FITC)–dextran, a non-absorbable high molecular weight molecule, in serum after oral administration as mentioned in previous publications (40). Serum BG was determined by Fungitell (Associates of Cape Cod, East Falmouth, MA, United States) and serum endotoxin (LPS) was measured by HEK-Blue LPS Detection (InvivoGen, San Diego, CA, United States). When values of BG and LPS at <7.8 and at <0.01 EU/ml, respectively, were beyond the lower range of the standard curve, data were recorded as 0.

### Liver Histology, Intestinal Cytokines, Fecal Fungal Burden, and Fecal pH

Paraffin-embedded liver sections (4 μm thick) stained by H&E from 10% formalin-fixed samples were evaluated with a scoring system of obesity-induced liver damage as the following: steatosis (0–3), lobular inflammation (0–3), and hepatocellular ballooning degeneration (0–2) (41). For intestinal cytokine detection, intestinal tissues were weighed, cut, thoroughly sonicated (15 s with pulse off 5 s for 5 times; High Intensity Ultrasonic Processor, Newtown, CT, United States) in 500 μl of ice-cold PBS containing protease inhibitor Cocktail (I3786; Sigma-Aldrich) and measured cytokines from the supernatant by ELISA (Invitrogen). For analysis of fungal burdens in feces, feces were suspended with PBS at a ratio of 100 μg per 1 μl and serially diluted before plating onto 0.1% chloramphenicol in Sabouraud Dextrose Agar (SDA; Thermo Scientific, Waltham, MA, United States) and aerobically incubated at 35°C for 72 h before colony enumeration. For fecal pH evaluation, 1 *g* of feces was thoroughly mixed with 2 ml of water before centrifugation at 4000 rpm for 3 min. Then, the pH of the supernatant was measured by a pH meter (Orion’4 star, pH Conductivity Benchtop; Thermo Scientific).

### Fecal Microbiome Analysis

Feces from nine mice (0.25 *g* per mouse) from different cages in each experimental group were divided into three samples per group (three mice per sample) before performing microbiota analysis. Total DNA from feces was extracted by GenUP gDNA extraction kit (Biotechrabbit, Germany) followed by 16S rDNA amplification for next-generation sequencing (NGS) with Illumina platform as previously published (42). For data analysis, the raw data were de-multiplexed by miSeq reporter software (version 2.6.2.3). Paired-end FASTQ sequences were then analyzed with QIIME2 pipeline (version 2018.8) (43). After that, joined reads were de-duplicated and clustered with 97% similarity by VSEARCH (44). Chimeric sequences were filtered out by UCHIME algorithm (45). The filtered reads were classified based on 99% operational taxonomic units (OTUs) clustered 16S Greengene database (2013.8) (46) using vsearch algorithm.

### Hepatocyte Cell-Line Experiments

HepG2, a human hepatoma cell line (ATCC HB-8065; Fisher Scientific), was maintained in Dulbecco’s Modified Eagle Medium (DMEM) with 10% fetal bovine serum (FBS), 1% penicillin/streptomycin antibiotics, and 1% sodium pyruvate in a humidified atmosphere of 5% CO_2_ at 37°C. HepG2 at 2 × 10^5^ cells/ml in a 96-well plate were incubated with or without 0.5 mM of palmitic acid (PA; Sigma-Aldrich), a saturated free fatty acid, in DMEM at 37°C for 48 h before further incubation with purified LPS (1 μg/ml) from *Escherichia coli* 026:B6 (Sigma-Aldrich) alone or in combination with CM-Pachyman (100 μg/ml; Megazyme, Bray, Ireland) as a representative of BG. After PA incubation for 24 h, intracellular lipid was determined by 0.3% Oil Red O solution (Sigma-Aldrich) and evaluated by Image J (NIH, Bethesda, MD, United States) in 10 randomized fields from each well as previously mentioned (47). Supernatant cytokine were determined using ELISA for human cytokines (TNF-α, IL-8, and IL-10; R&D Systems, Minneapolis, MN, United States). In addition, a neutral soluble glucan, a competitive Dectin-1 binding agent (at 150 μg/ml; InvivoGen), was incubated simultaneously with BG as a Dectin-1 inhibitor to explore the impact of Dectin-1, a BG receptor, on hepatocytes. Moreover, energy metabolism profiles of hepatocytes activated by PA simultaneously with LPS or with LPS + BG with glycolysis estimation through extracellular acidification rate and mitochondrial oxidative phosphorylation by oxygen consumption rate were performed using Seahorse XFp Analyzers (Agilent, Santa Clara, CA, United States) upon HepG2 at 1 × 10^4^ cells/well (47, 48).

### Macrophage Cell-Line Experiments

RAW264.7, a mouse macrophage cell line, at 1 × 10^5^ cells per well was incubated with 0.2 mM PA (Sigma-Aldrich) alone or in combination with LPS (1 μg/ml; Sigma-Aldrich) or BG, CM-Pachyman (100 μg/ml; Megazyme), or LPS + BG, similar to hepatocyte experiments, for 6 h before determination of Oil Red O staining. In parallel, supernatant cytokines were measured by ELISA for mouse cytokines (TNF-α, IL-6, and IL-10; Invitrogen). In addition, a Dectin-1 inhibitor (150 μg/ml; InvivoGen) was incubated with BG to explore the impact of Dectin-1 in macrophages.

### Statistical Analysis

Mean ± SE was used for data presentation. The differences between groups were examined for statistical significance by one-way ANOVA followed by Tukey’s analysis or Student’s *t-*test for comparisons of multiple groups or two groups, respectively. Survival analysis was performed by log-rank test. All statistical analyses were performed with SPSS 11.5 software (SPSS, IL, United States) and GraphPad Prism version 7.0 software (La Jolla, CA, United States). A *p* value of < 0.05 was considered statistically significant.

## Results

**FIGURE 2:**
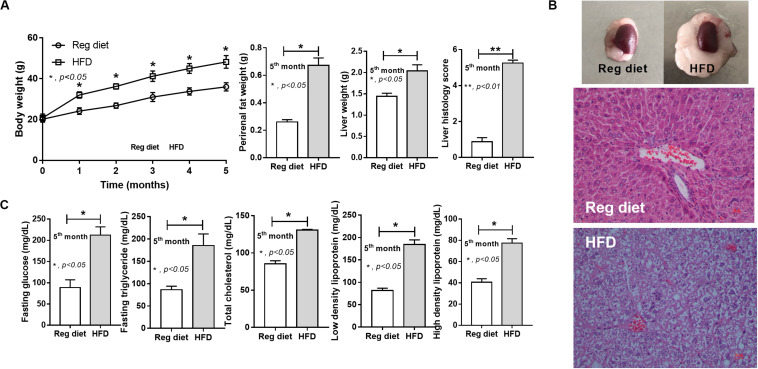
Characteristics of mice fed with a regular diet and a high-fat diet (HFD) as demonstrated by body weight, peri-renal fat weight, liver weight, and liver histology score (**A**; *n* = 7–8/time point and *n* = 6–8/group) with representative pictures of peri-renal fat and H&E-stained liver histology **(B)** together with metabolic parameters (**C**; *n* = 6–8/group) are demonstrated. *p < 0.05; **p < 0.01.

As expected, HFD-induced obesity in mice led to increased body weight, peri-renal fat, liver weight, fatty liver score, fasting blood glucose, and altered lipid profiles (Figure 2).

### Sepsis Severity of Obese Mice With and Without *Candida* Administration, an Impact of Gut-Permeability Defect and Gut Dysbiosis

**FIGURE 3:**
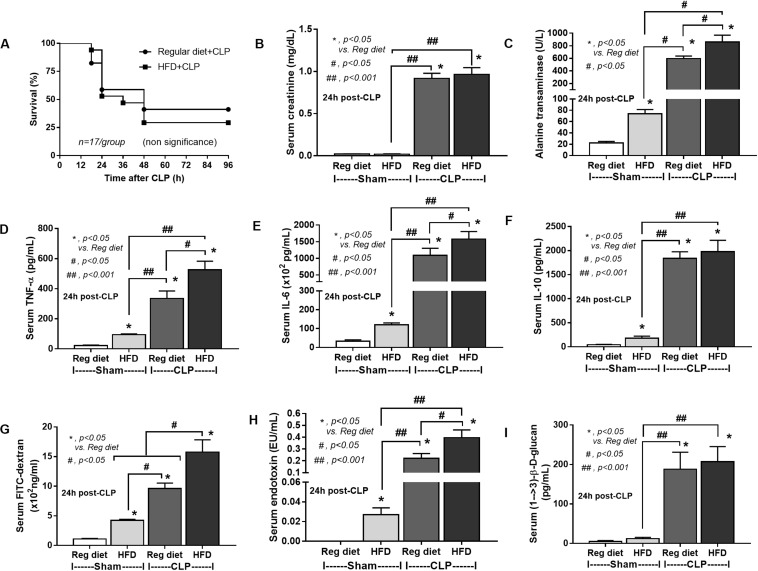
Characteristics of mice fed a regular diet or a high-fat diet (HFD) with sham or cecal ligation and puncture (CLP) surgery as determined by survival analysis **(A)**, kidney and liver injury **(B,C)**, serum cytokines **(D–F)**, gut leakage by FITC–dextran **(G)**, endotoxemia **(H)**, and serum (1→3)-β-D-glucan (**I**; *n* = 6–8/group for **B–I**) are demonstrated. *p < 0.05; ^#^*p* < 0.05; ^##^*p* < 0.001.

**FIGURE 4:**
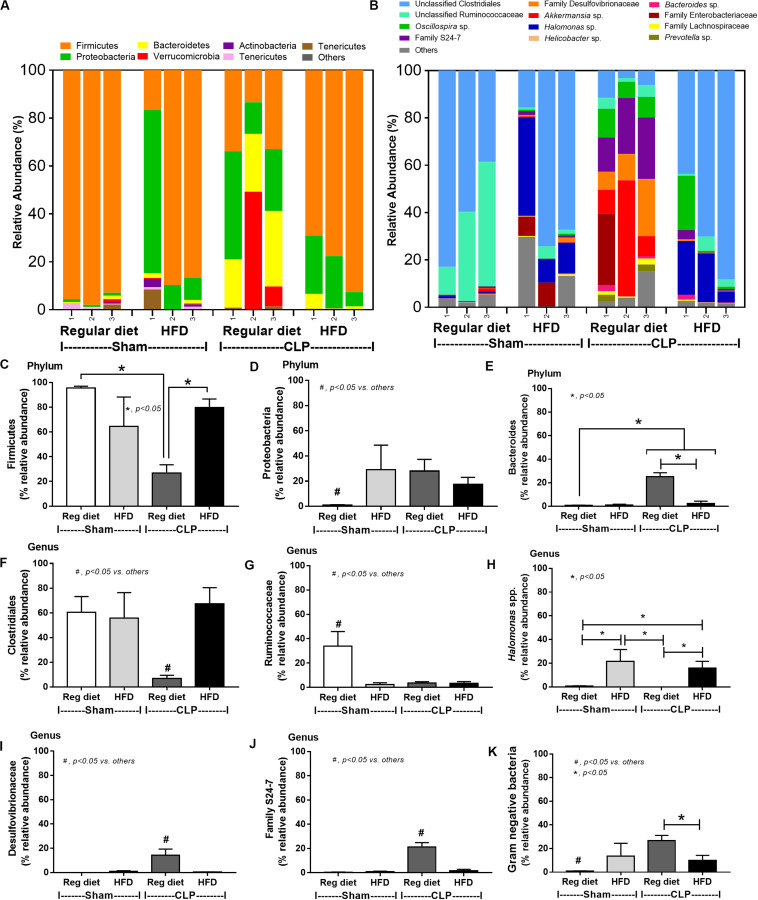
Gut microbiota analysis from feces of mice fed with a regular diet or a high-fat diet (HFD) with sham or cecal ligation and puncture (CLP) surgery by relative abundance of bacterial diversity at phylum **(A)** and at genus **(B)** with the better visualization **(C–J)** and abundance of total Gram-negative bacteria determined from phylum **(K)** are demonstrated. *p < 0.05; ^#^*p* < 0.05.

**FIGURE 5:**
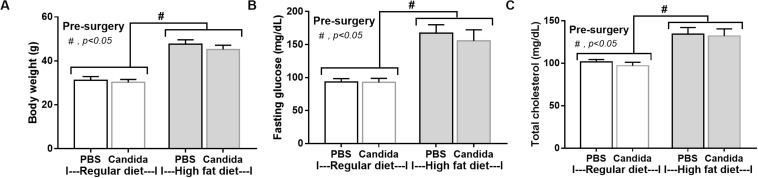
Characteristics of mice fed with a regular diet or a high-fat diet (HFD) treated with phosphate buffer solution (PBS) or *Candida* at 5th month of the experiments as determined by body weight **(A)**, fasting glucose and total cholesterol in blood (**B,C**; *n* = 6–8/group) are demonstrated. ^#^*p* < 0.05.

**FIGURE 6:**
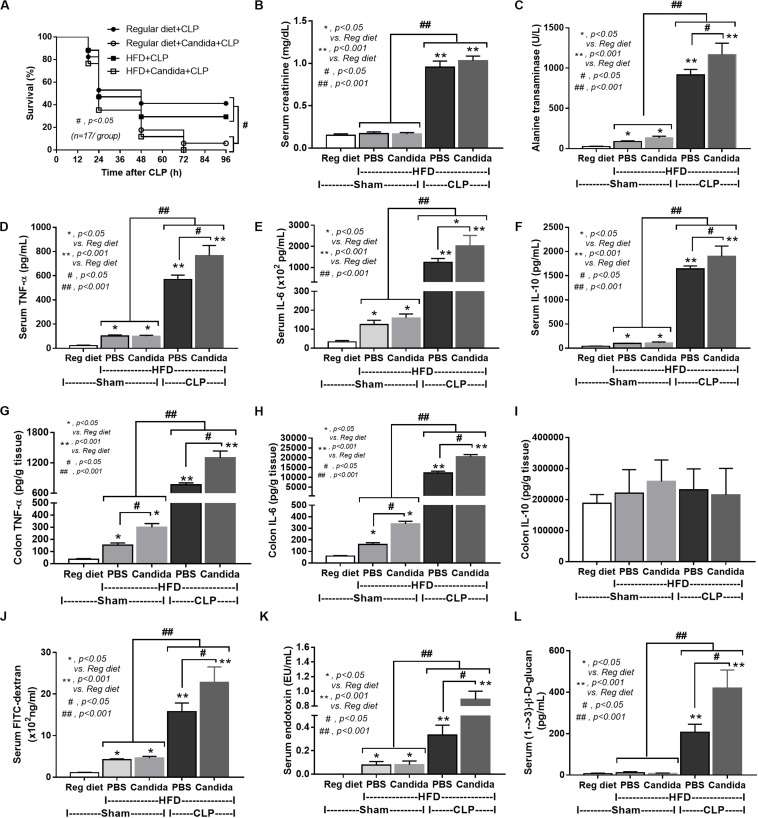
Characteristics of mice fed a regular diet that subjected to sham surgery and mice fed a high-fat diet (HFD) treated with phosphate buffer solution (PBS) or *Candida* that were subjected to sham or cecal ligation and puncture (CLP) as determined by survival analysis **(A)**, kidney and liver injury **(B,C)**, serum cytokines **(D–F)**, colon cytokine **(G–I)**, gut leakage by FITC–dextran **(J)**, endotoxemia **(K)**, and serum (1→3)-β-D-glucan (**L**; *n* = 6–8/group) are demonstrated. *p < 0.05; **p < 0.001; ^#^*p* < 0.05; ^##^*p* < 0.001.

In mice subjected to CLP and fed a HFD, but not treated with *Candida*, sepsis were more severe than regular diet mice as determined by survival, organ injury (serum creatinine and alanine transaminase), serum cytokines (TNF-α and IL-6), gut leakage by FITC–dextran and endotoxemia, but not serum BG (Figures 3A–I). In mice that were not subjected to CLP, there were slightly elevated serum endotoxin (Figure 3H) along with gut dysbiosis in HFD mice compared with regular diet mice as demonstrated by fecal total Gram-negative bacteria (Proteobacteria in *Halomonas* spp.) and reduced Ruminococcaceae, beneficial cellulolytic Gram-positive anaerobes (49) (Figure 4). Mice in the HFD-CLP group that were not treated with *Candida* demonstrated lower total fecal Gram-negative bacteria (Desulfovibrionaceae, Bacteroides) with higher Firmicutes, beneficial Gram-positive anaerobes, and Clostridiales family, a group of bacteria including mucosal invasive *Clostridium* spp. (50), in comparison with mice in the regular diet–CLP group (Figure 4). On the other hand, *Candida* administration did not induce diarrhea (data not shown) and did not alter obesity parameters (Figure 5), but it enhanced CLP mortality in both regular diet and HFD mice (Figure 6A).

**FIGURE 7:**
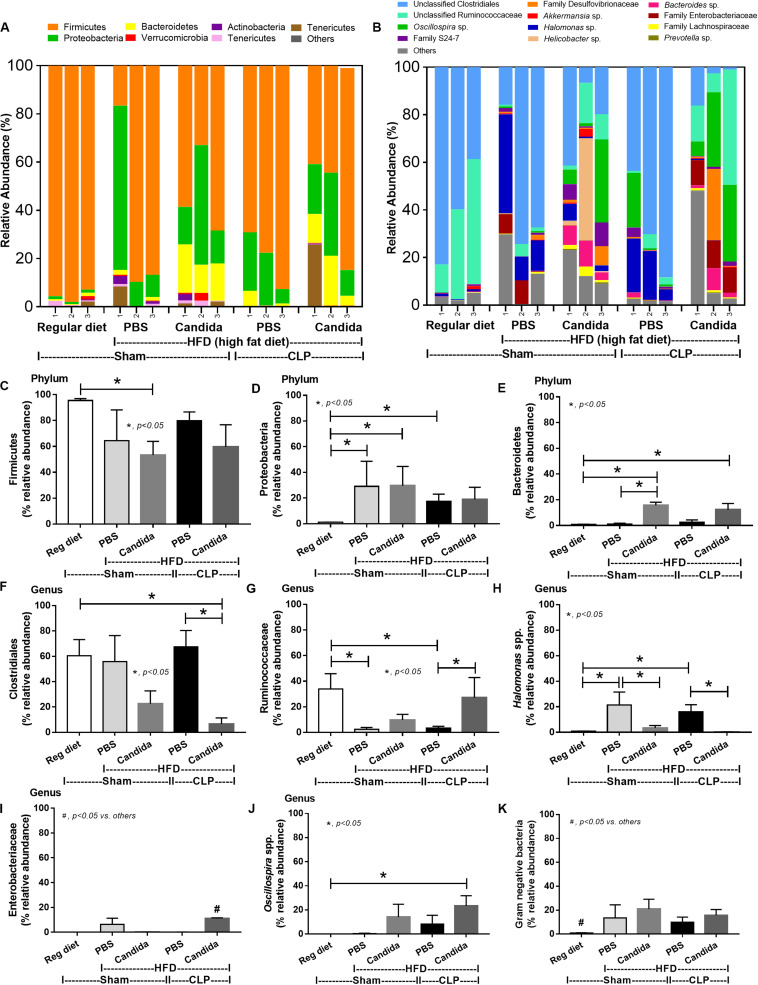
Gut microbiota analysis from feces of mice fed a regular diet that were subjected to sham surgery and mice fed a high-fat diet (HFD) treated with phosphate buffer solution (PBS) or *Candida* that were subjected to sham or cecal ligation and puncture (CLP) by relative abundance of bacterial diversity at phylum **(A)** and at genus **(B)** with the better visualization **(C–J)** and abundance of total Gram-negative bacteria determined from phylum **(K)** are demonstrated. *p < 0.05; ^#^*p* < 0.05.

In addition, mice in the HFD-CLP group treated with *Candida* exhibited more severe sepsis as determined by increased mortality, liver injury, serum cytokines, colon inflammation, gut leakage, endotoxemia, and glucanemia, but not serum creatinine when compared with mice in the HFD-CLP group that were not treated with *Candida* (Figures 6A–L). In mice fed a HFD but not subjected to CLP, *Candida* did not worsen obesity-induced liver injury, gut leakage, and serum cytokines, but it did activate local inflammation (colon TNF-α and IL-6; Figures 6C–H) without diarrhea when compared with mice in the HFD group that were not treated with *Candida*. This implies healthy mucosal barriers in non-CLP mice. Without sepsis, there were non-different fecal total Gram-negative bacteria (increased Bacteroides but decreased *Halomonas* spp.; Figure 7) and gut leakage (Figure 6I) when comparing between HFD mice with versus without *Candida*.

Of note, mice fed a HFD but not subjected to CLP-induced dysbiosis were demonstrated by increased total fecal Gram-negative bacteria (Proteobacteria and *Halomonas* spp.) with reduced beneficial Gram-positive anaerobes (Ruminococcaceae) when compared with mice in the regular diet group that were not treated with *Candida* (Figure 7). Mice in the HFD-CLP group treated with *Candida* demonstrated no change in total Gram-negative bacteria with increased Enterobacteriaceae, pathogenic Gram-negative aerobes (51, 52) in comparison with mice in the HFD-CLP group that were not treated with *Candida* (Figure 7). Only slight alterations in bacterial diversity were demonstrated between HFD versus regular diet with CLP (non-*Candida*) and between HFD with versus without *Candida* (Supplementary Figures 1A–F).

### Additive Effect Between Endotoxin and (1→3)-β-D-Glucan Toward Hepatocytes and Macrophages

**FIGURE 8:**
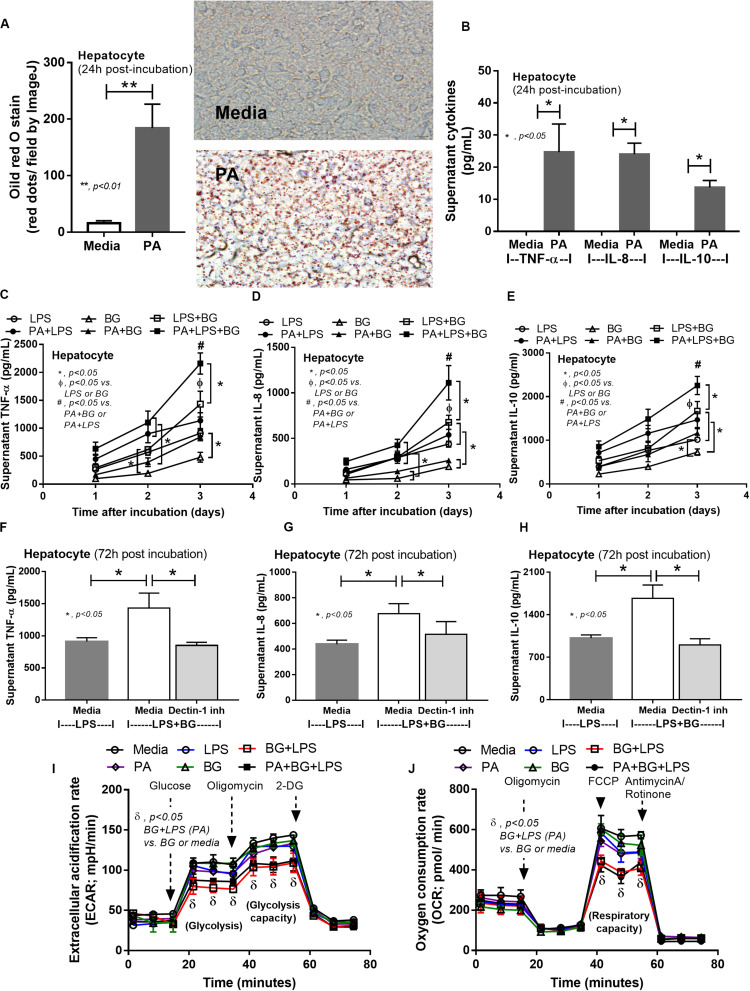
Intracellular lipid accumulation by Oil Red O staining with representative pictures and supernatant cytokines in HepG2 cells (hepatocytes) after activation by palmitic acid (PA), a representative saturated fatty acid, or media control **(A,B)**, supernatant cytokines from PA-activated hepatocytes with endotoxin (LPS), (1→3)-β-D-glucan (BG), or LPS plus BG (LPS + BG; **C–E**), supernatant cytokines with or without Dectin-1 inhibitor **(F–H)**, and extracellular flux analysis pattern at 48 h of several activations **(I,J)** are demonstrated. 2-DG, 2-Deoxy-D-glucose; FCCP, carbonyl cyanide-4-(trifluoromethoxy) phenylhydrazone (independent triplicate experiments were performed). *p < 0.05; **p < 0.01; ^#^*p* < 0.05.

**FIGURE 9:**
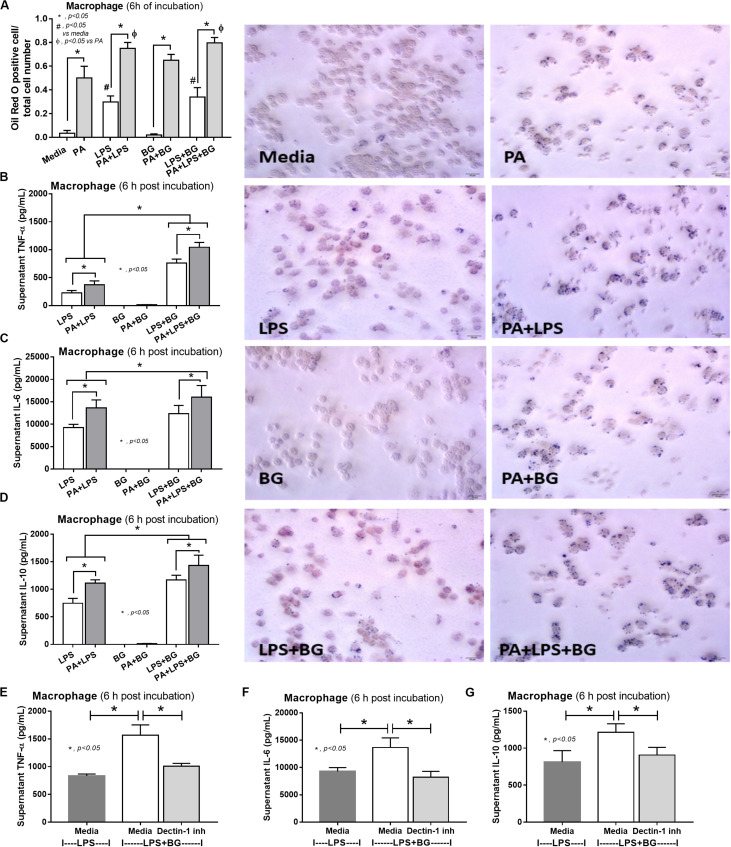
Characteristics of RAW264.7 cells (macrophages) after 6-h incubation of palmitic acid (PA), a representative saturated fatty acid, or media control together with endotoxin (LPS), (1→3)-β-D-glucan (BG), or LPS plus BG (LPS + BG) as determined by intracellular lipid accumulation by Oil Red O staining with representative pictures **(A)**, supernatant cytokines **(B–D)**, and supernatant cytokines with or without Dectin-1 inhibitor **(E–G)** are demonstrated (independent triplicate experiments were performed). *p < 0.05; ^#^*p* < 0.05.

To provide mechanistic data for the previously described phenomena, we studied the interactions of LPS, BG, and PA on inflammation (53, 54) and mitochondrial function in hepatocytes (HepG2) and macrophages (RAW264.7 cells) (31). As such, PA, a representative saturated fatty acid, induced lipid accumulation, mild cytokine production, and amplified cytokine responses in HepG2 cells after stimulation with LPS plus BG (LPS + BG; Figures 8A–E). Supernatant cytokines of LPS + BG activated hepatocytes were suppressed by Dectin-1 inhibitor (Figures 8F–H) implying Dectin-1-dependent signaling. Although BG activation with or without PA induced only mild cytokine responses, BG was an effective adjuvant for LPS stimulation as LPS + BG induced higher cytokine production compared with LPS alone (Figures 8C–E). In addition, the separated activation by LPS, BG, or PA in HepG2 cells showed a tendency of reduced mitochondrial respiration compared with media control, but it did not reach a significant level (Figures 8I,J). Meanwhile, LPS + BG significantly reduced glycolysis capacity (glycolysis activity during mitochondrial cessation) and respiratory capacity (mitochondria activity during glycolysis blocking) compared with media control (Figures 8I,J). However, an addition of PA into LPS + BG could not alter hepatocyte energy metabolism when compared with LPS + BG (Figures 8I,J). In macrophages, PA enhanced lipid accumulation and increased supernatant cytokines of LPS or LPS + BG activation when compared with the conditions without PA (Figures 9A–D). The activation by PA + LPS + BG in macrophages demonstrated the most prominent cytokine responses (Figures 9A–D). Furthermore, Dectin-1 inhibitor reduced macrophage responses against LPS + BG (Figures 9E–G). These data support the possible systemic inflammatory effect of LPS and BG from gut translocation against both hepatocytes and macrophages.

### Probiotic Attenuates Sepsis Severity in Obese Mice, Regardless of *Candida* Administration

**FIGURE 10:**
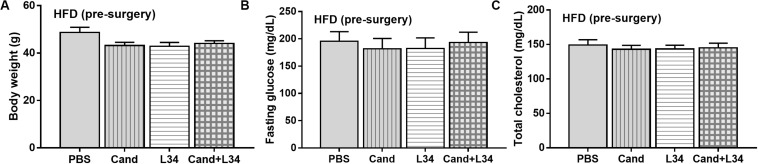
Characteristics of mice fed a high-fat diet (HFD) treated with phosphate buffer solution (PBS) or *Candida* with or without *Lactobacillus rhamnosus* L34 (L34) at 5th month of the experiments (before cecal ligation and puncture operation) as determined by body weight **(A)**, fasting glucose and total cholesterol in blood (**B,C**; *n* = 6–8/group) are demonstrated.

**FIGURE 11:**
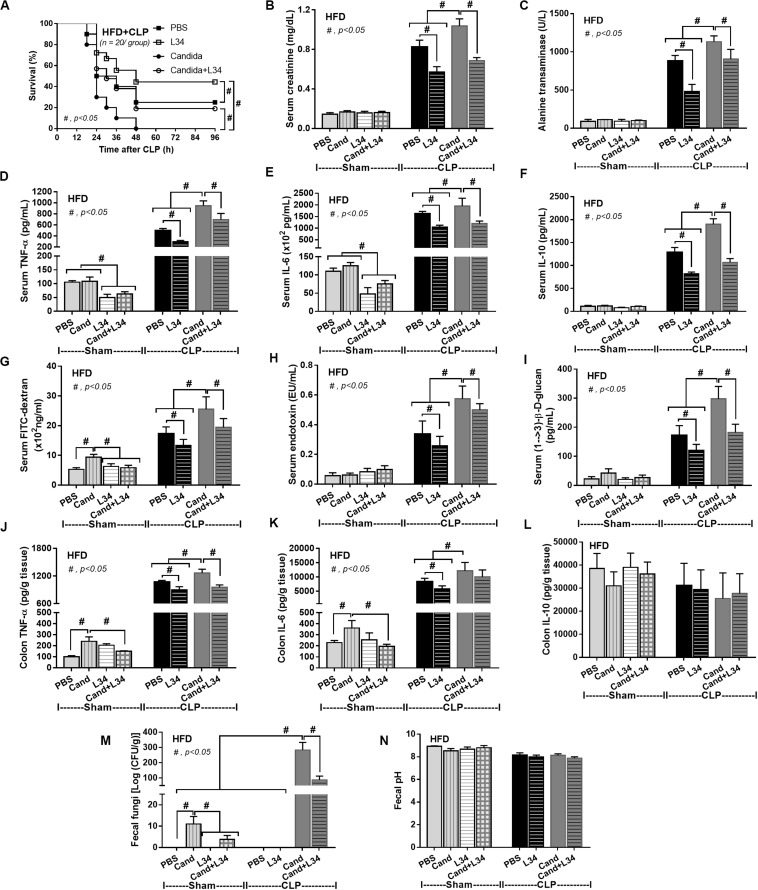
Characteristics of mice fed a high-fat diet (HFD) treated with phosphate buffer solution (PBS) or *Candida* with or without *Lactobacillus rhamnosus* L34 (L34) that were subjected to sham or cecal ligation and puncture (CLP) surgery as determined by survival analysis **(A)**, kidney and liver injury **(B,C)**, serum cytokines **(D–F)**, gut leakage by FITC–dextran **(G)**, endotoxemia **(H)**, serum (1→3)-β-D-glucan **(I)**, cytokines from ascending colon **(J–L)**, fecal fungal burdens and fecal pH (**M, N**; *n* = 6–8/group for **B–N**) are demonstrated. ^#^*p* < 0.05.

**FIGURE 12:**
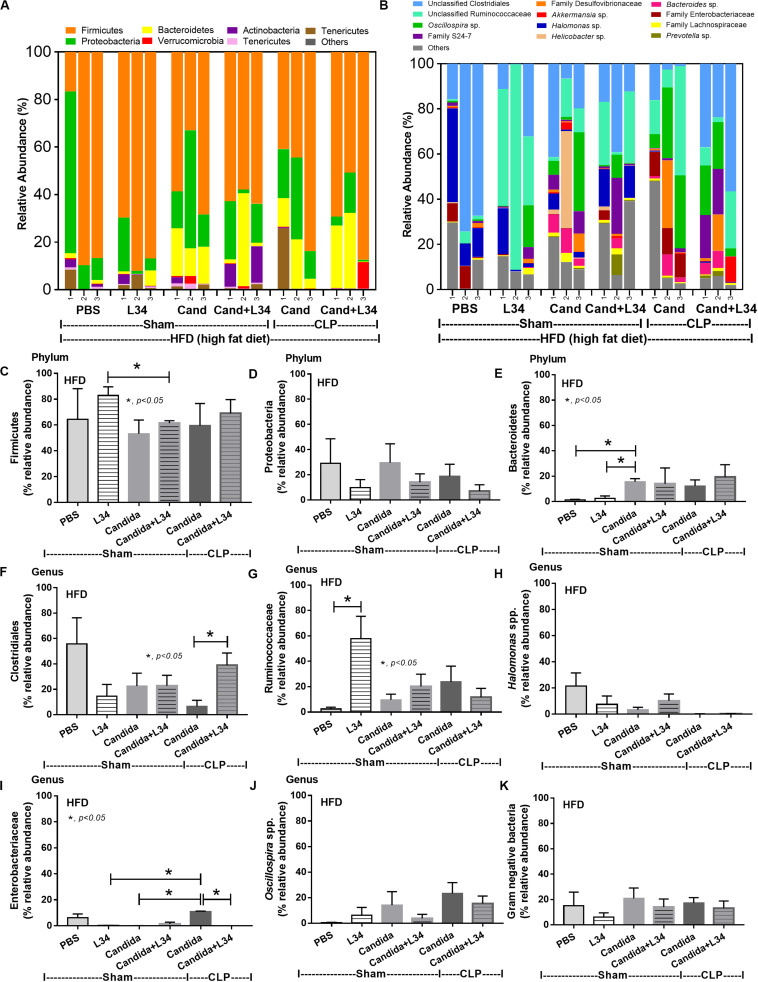
Gut microbiota analysis from feces of mice fed a high-fat diet (HFD) treated with phosphate buffer solution (PBS) or *Candida* with or without *Lactobacillus rhamnosus* L34 (L34) that were subjected to sham or cecal ligation and puncture (CLP) surgery by relative abundance of bacterial diversity at phylum **(A)** and at genus **(B)** with better visualization **(C–J)** and abundance of total Gram-negative bacteria determined from phylum **(K)** are demonstrated. *p < 0.05.

**FIGURE 13:**
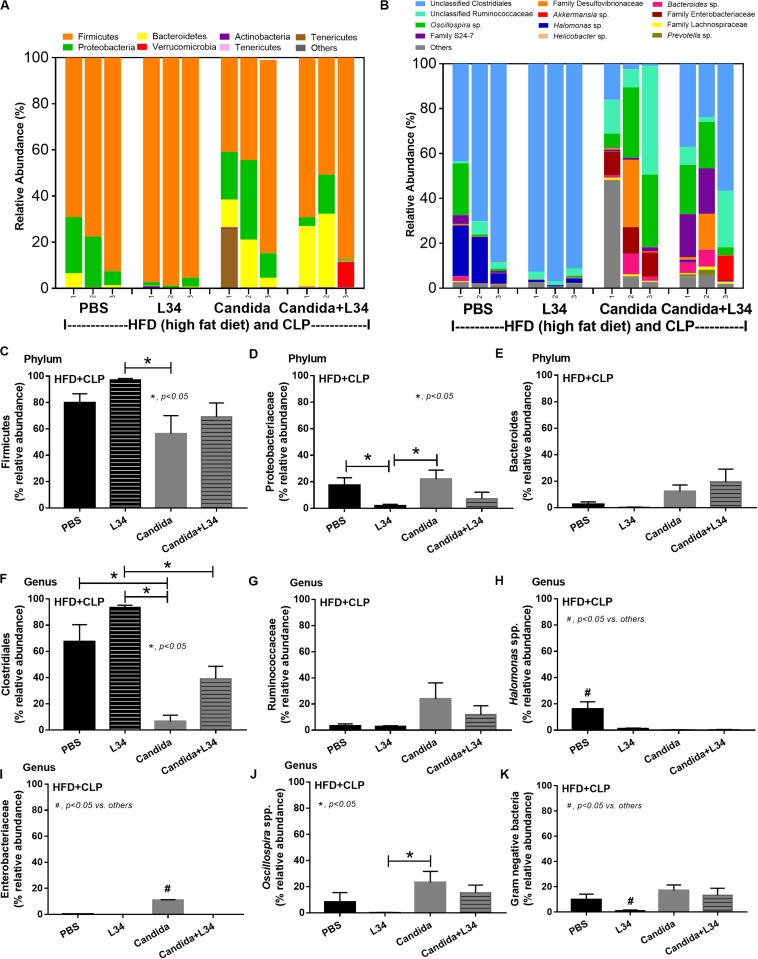
Gut microbiota analysis from feces of mice fed a high-fat diet (HFD) treated with phosphate buffer solution (PBS) or *Candida* with or without *Lactobacillus rhamnosus* L34 (L34) that were subjected to cecal ligation and puncture (CLP) surgery by relative abundance of bacterial diversity at phylum **(A)** and at genus **(B)** with the better visualization **(C–J)** and abundance of total Gram-negative bacteria determined from phylum **(K)** are demonstrated. *p < 0.05; ^#^*p* < 0.05.

Although L34 neither induced diarrhea (data not shown) nor improved obesity complications (Figure 10), L34 attenuated CLP severity in HFD mice regardless of *Candida* administration as determined by survival, organ injury, serum cytokines, colon inflammation, gut leakage, and fecal fungal burdens (Figures 11A–L) partly through amelioration of gut dysbiosis. Accordingly, in mice fed a HFD-*Candida* but not subjected to CLP, L34 reduced fecal fungal burdens (Figure 11M) and increased Ruminococcaceae bacteria, a beneficial short-chain fatty acid–producing bacterial group (55, 56), without an effect on total fecal Gram-negative bacteria (Figure 12). In the HFD-CLP group that were not treated with *Candida*, L34 reduced total Gram-negative bacteria in feces, especially Proteobacteria in *Halomonas* spp. (Figure 13). On the other hand, L34 reduced fecal fungi and Enterobacteriaceae bacteria, pathogenic Gram-negative aerobe, without an effect on total fecal Gram-negative bacteria in the HFD-CLP group treated with *Candida* (Figure 13). However, L34 did not alter bacterial diversity index (Supplementary Figures 1G–L). Of note, the rarefaction curves are demonstrated in the microbiome analysis data (Supplementary Figure 1M).

## Discussion

Because *C. albicans* in mouse feces are detectable only by PCR (10), but not by culture (25) and differs from the human condition (26), the influence of *C. albicans* is evaluated through *C. albicans* administration. Here, *Candida* pretreatment in obese mice worsened sepsis through enhanced systemic inflammation induced by LPS and BG from gut translocation which implies the importance of gut fungi toward sepsis in obesity.

### Impact on Gut Leakage and Gut Dysbiosis of *Candida* in Sepsis-Obese Mice

Endotoxemia (57) in obesity (without sepsis) as a result of HFD increased fecal Gram-negative bacteria (58, 59) that is enhanced by sepsis-induced gut leakage has been previously mentioned (60–62). Here, several patterns of bacterial dysbiosis in obese mice in comparison with regular-diet mice were demonstrated including (1) increased total Gram-negative bacteria in mice fed a HFD without *Candida* and not subjected to CLP (Figure 4K), (2) increased Bacteroides, Gram-negative anaerobes in several pathogenic conditions (63), in mice fed a HFD-*Candida* but not subjected to CLP (Figure 7E), (3) increased pathogenic bacteria (Clostridiales) in HFD-CLP mice that were not treated with *Candida* (Figure 4F), and (4) increased mucosal-invasive pathogenic bacteria, Enterobacteriaceae (49, 52, 64), in HFD-CLP mice treated with *Candida*. In addition, CLP also increased *Candida* burdens in feces compared with CLP non-*Candida* and supported the impact of mucosal-immunity defect in sepsis (17, 65–67). Although *Candida* gavage in healthy mice did not increase fecal fungi, gut *Candida* induced local gut inflammation without gut leakage. Hence, intestinal *Candida* could enhance gut leakage in obese-sepsis mice from both direct *Candida* mucosal damage and indirect injury through *Candida*-induced bacterial gut dysbiosis.

### Enhanced Inflammatory Responses of *Candida* in Sepsis-Obese Mice and Role of Saturated Fatty Acid

During gut leakage, intestinal *Candida* increases BG in gut contents that could be delivered to the liver and lymphatic system (31). In hepatocytes, an additive inflammatory effect of LPS was amplified by BG through the activation on Dectin-1, a receptor for BG, as the amplification was neutralized by a Dectin-1 inhibitor. In addition, LPS + BG, but not in separation, reduced the capacity of glycolysis and mitochondria function in hepatocytes which might be associated with a significant hepatocyte injury (68, 69). It is interesting to note that the property of LPS and BG from different organisms might be different. This includes the quantity of lipid A, an LPS conserved lipid region with the pro-inflammatory property (70, 71) and BG molecular size (72). Here, LPS and BG from *E. coli* and Pachyman, respectively, were used as proof-of-concept experiments which might be different from other representative molecules. There has been a previous report that LPS *E. coli* K12 significantly reduce mitochondrial function in HEPG2 cells (73). Meanwhile, LPS *E. coli* 026:B6 in our experiments showed only a tendency of reduction. Despite this limitation, LPS + BG altered cytokine responses and cell energy metabolism in hepatocytes enhanced by saturated fatty acid. This supports HFD-induced metabolic pro-inflammation (53, 54, 74). Saturated fatty acid alone did not alter cell energy metabolism of hepatocytes. Moreover, additive effects of BG on LPS that are enhanced by saturated fatty acids have been also observed in macrophages in the current study and in other publications (22–24, 75). However, extracellular flux analysis in macrophages was not performed here due to well-known LPS-enhanced glycolysis (76). Our data support that saturated fatty acids, which are absorbed through portal vein (77), enhances LPS activity in hepatocytes and macrophages (53, 54) and induced cytokine production (78). This suggests the inflammatory aggravating property of dietary saturated fatty acids on LPS + BG in sepsis with obesity.

### Probiotic Treatment in Sepsis, the Attenuation of Gut Dysbiosis, and Gut Leakage

Administration of L34 attenuated sepsis severity in obese mice with and without *Candida*, at least in part, through the reduced severity of gut leakage and gut dysbiosis. Here, several patterns of the attenuation of gut dysbiosis by L34 were demonstrated including (1) increased Ruminococcaceae which is a beneficial butyrate (short-chain fatty acid)–producing bacterial group (55, 56), in mice fed a HFD without *Candida* but not subjected to CLP; (2) reduced fecal Gram-negative bacteria which are a source of LPS in gut contents in HFD-CLP mice that were not treated with *Candida*; and (3) reduced pathogenic Enterobacteriaceae (79) and fungi in HFD-CLP mice treated with *Candida*. In translation, manipulation of gut leakage and/or fungal burdens by probiotics should be one interesting strategy against sepsis in obesity. However, several limitations on the similarity to patient obesity should be mentioned: (1) ingestion of 60% saturated fat diet is higher than most of the regular diets in human (80), (2) oral gavage also induced stress that might different from obesity in patients (81, 82), and (3) the dose of probiotics that is equivalent to human weight is 2 × 10^12^ CFU/dose that possibly induces some adverse effects (83). More studies in patients are warranted.

**FIGURE 14:**
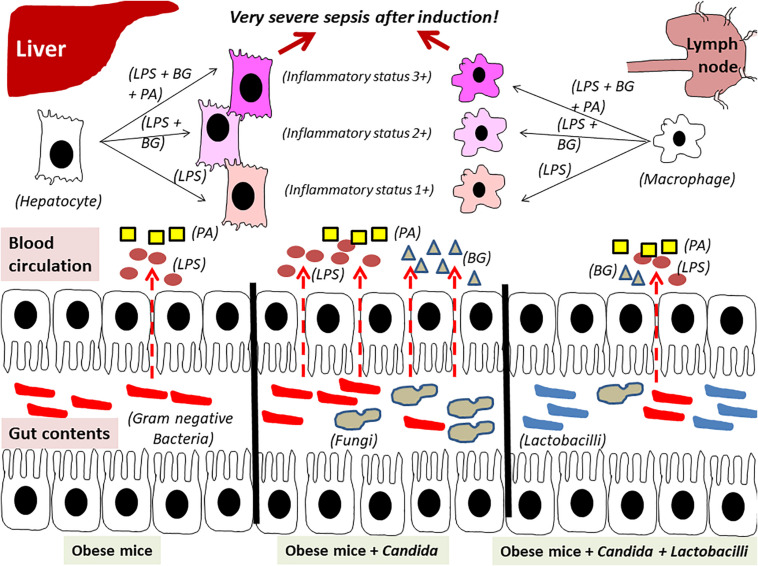
The proposed hypothesis demonstrates gut leakage in mice fed a high-fat diet (HFD) treated with *Candida* is more severe than mice fed a HFD that were not treated with *Candida* due to prominent gut translocation of lipopolysaccharide (LPS) and (1→3)-β-D-glucan (BG), a major cell wall component of Gram-negative bacteria and *Candida*, respectively, that are delivered to liver and systemic circulation (31). Additive effect of LPS with BG (LPS + BG) on hepatocytes and macrophages is amplified by palmitic acid (PA), a pro-inflammatory saturated fatty acid, resulting in higher inflammatory status that enhances sepsis severity. Meanwhile, *Lactobacilli* spp. attenuate gut dysbiosis, gut leakage, systemic inflammation, and sepsis severity (dotted line is gut translocation of LPS and BG).

In conclusion, obesity and *Candida* administration enhanced sepsis severity through gut dysbiosis–induced gut leakage and saturated fatty acid–amplified pathogen-associated molecules induced inflammation which could be attenuated by probiotics (Figure 14).

## Data Availability Statement

The raw data supporting the conclusions of this article will be made available by the authors, without undue reservation, to any qualified researcher.

## Ethics Statement

The animal study was reviewed and approved by The Institutional Animal Care and Use Committee of the Faculty of Medicine, Chulalongkorn University, Bangkok, Thailand (SST 04/2561).

## Author Contributions

WP designed and coordinated all the experiments, performed *in vitro* and *in vivo* experiments, and wrote the manuscript and approved. VS performed microbiome analysis and approved the manuscript. PC performed microbiome analysis and approved the manuscript. TO performed *in vitro* experiments and approved the manuscript. CD performed *in vitro* experiments and approved the manuscript. SP supervised microbiome analysis and approved the manuscript. ST supervised the *in vitro* experiment and also provided the probiotic in this study. AL designed and coordinated all the experiments, analyzed all of these experiment, and wrote the manuscript and approved. All authors contributed to the article and approved the submitted version.

## Conflict of Interest

The authors declare that the research was conducted in the absence of any commercial or financial relationships that could be construed as a potential conflict of interest.

## Supplementary Material

Supplementary Figure 1 Alpha diversity by Chao 1 richness estimation and Shannon evenness, analysis with β-diversity plot of mice in different groups, including regular diet or high-fat diet (HFD) after sham or cecal ligation and puncture (CLP; **A–C)**, sham regular diet versus sham or CLP of HFD with *Candida* or phosphate buffer solution (PBS; **D–F)**, sham or CLP in HFD with PBS, *Lactobacillus rhamnosus* L34 (L34), *Candida* with or without L34 **(G–I)**, CLP and HFD (HFD + CLP) with PBS or *Candida* with or without L34 **(J–L)**, and rarefaction curves of microbiome analysis **(M)** are demonstrated. OTUs, operational taxonomic units; PC, principal component.
