# Corrigendum: *Candida* Administration Worsens Cecal Ligation and Puncture-Induced Sepsis in Obese Mice Through Gut Dysbiosis Enhanced Systemic Inflammation, Impact of Pathogen-Associated Molecules From Gut Translocation and Saturated Fatty Acid

**DOI:** 10.3389/fimmu.2020.613095

**Published:** 2020-11-05

**Authors:** Wimonrat Panpetch, Vorthon Sawaswong, Prangwalai Chanchaem, Thunnicha Ondee, Cong Phi Dang, Sunchai Payungporn, Somying Tumwasorn, Asada Leelahavanichkul

**Affiliations:** ^1^Department of Microbiology, Faculty of Medicine, Chulalongkorn University, Bangkok, Thailand; ^2^Program in Bioinformatics and Computational Biology, Graduate School, Chulalongkorn University, Bangkok, Thailand; ^3^Department of Biochemistry, Faculty of Medicine, Chulalongkorn University, Bangkok, Thailand; ^4^Center of Excellence in Systems Biology, Chulalongkorn University, Bangkok, Thailand; ^5^Medical Microbiology, Interdisciplinary Program, Graduate School, Chulalongkorn University, Bangkok, Thailand; ^6^Translational Research in Inflammation and Immunology Research Unit, Department of Microbiology, Chulalongkorn University, Bangkok, Thailand

**Keywords:** intestinal *Candida*, obesity, high-fat diet, probiotics, cecal ligation and puncture, dysbiosis, gut leakage

## Addition of an Author

Somying Tumwasorn was not included as an author in the published article. The corrected Author Contributions Statement appears below.

## Author Contributions

WP designed and coordinated all the experiments, performed in vitro and in vivo experiments, and wrote the manuscript and approved. VS performed microbiome analysis and approved the manuscript. PC performed microbiome analysis and approved the manuscript. TO performed in vitro experiments and approved the manuscript. CD performed in vitro experiments and approved the manuscript. SP supervised microbiome analysis and approved the manuscript. ST supervised the in vitro experiment and also provided the probiotic in this study. AL designed and coordinated all the experiments, analyzed all of these experiment, and wrote the manuscript and approved. All authors contributed to the article and approved the submitted version.

The authors apologize for this error and state that this does not change the scientific conclusions of the article in any way. The original article has been updated.

