# Supplementary figures and images for: Candida Administration Worsens Cecal Ligation and Puncture-Induced Sepsis in Obese Mice Through Gut Dysbiosis Enhanced Systemic Inflammation, Impact of Pathogen-Associated Molecules From Gut Translocation and Saturated Fatty Acid
Source: Front Immunol. 2020 Sep 25;11:561652. doi: 10.3389/fimmu.2020.561652 (PMC7545113; doi:10.3389/fimmu.2020.561652)

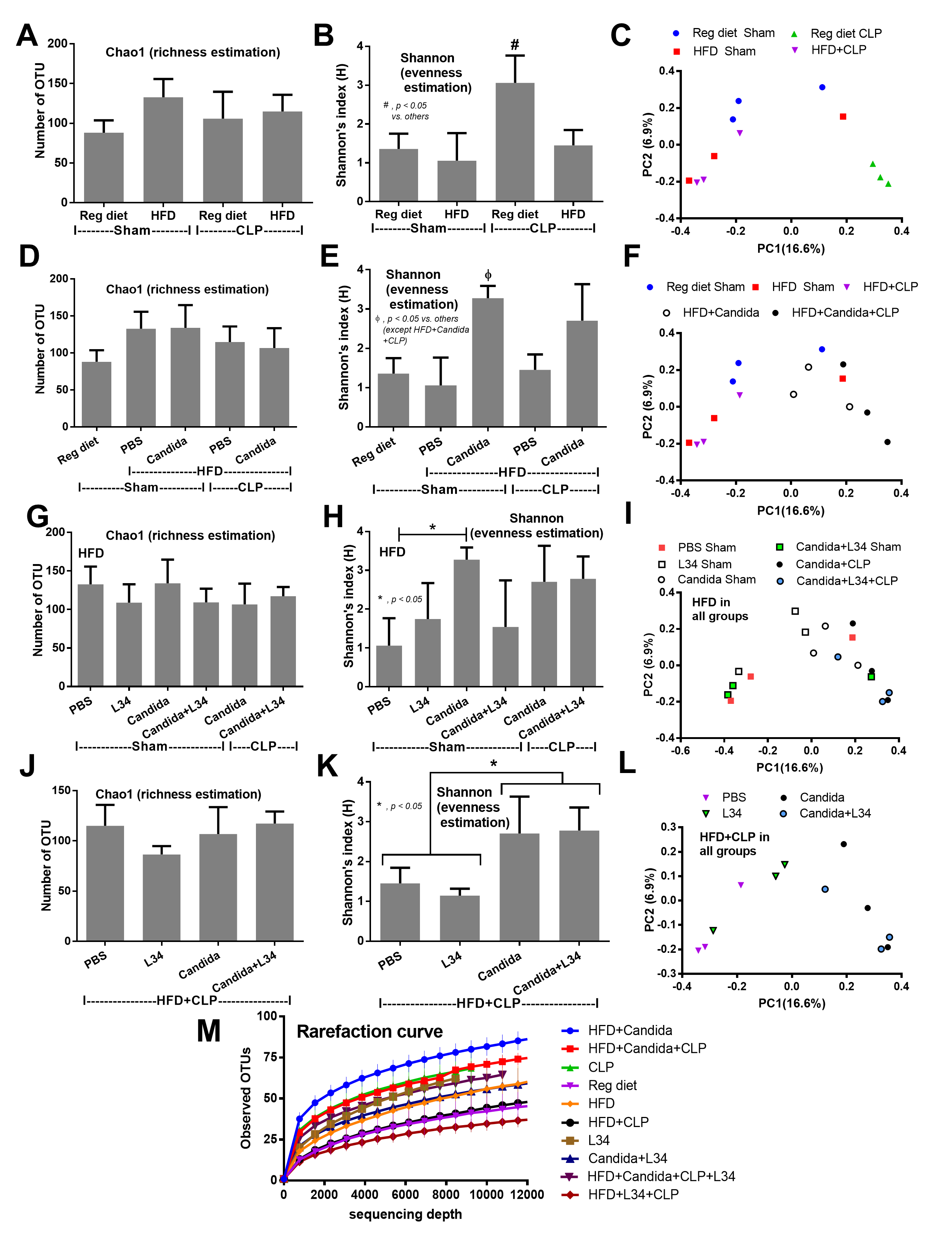

Supplement: Supplementary Figure 1 — Alpha diversity by Chao 1 richness estimation and Shannon evenness, analysis with β-diversity plot of mice in different groups, including regular diet or high-fat diet (HFD) after sham or cecal ligation and puncture (CLP; A–C), sham regular diet versus sham or CLP of HFD with Candida or phosphate buffer solution (PBS; D–F), sham or CLP in HFD with PBS, Lactobacillus rhamnosus L34 (L34), Candida with or without L34 (G–I), CLP and HFD (HFD + CLP) with PBS or Candida with or without L34 (J–L), and rarefaction curves of microbiome analysis (M) are demonstrated. OTUs, operational taxonomic units; PC, principal component. [file Image_1.TIF]
